# Stage 4 neuroblastoma: sequential hemi-body irradiation or high-dose chemotherapy plus autologous haemopoietic stem cell transplantation to consolidate primary treatment

**DOI:** 10.1038/sj.bjc.6602615

**Published:** 2005-05-17

**Authors:** R Luksch, M Podda, L Gandola, D Polastri, L Piva, R Castellani, P Collini, M Massimino, G Cefalo, M Terenziani, A Ferrari, M Casanova, F Spreafico, C Meazza, F Bozzi, A Marchianò, F Ravagnani, F Fossati-Bellani

**Affiliations:** 1 Unità di Pediatria, Istituto Nazionale Tumori di Milano, Via Venezian, 1-20133 Milan, Italy; 2 Unità di Radioterapia, Istituto Nazionale Tumori di Milano, Via Venezian, 1-20133 Milan, Italy; 3 Unità di Chirurgia Urologica, Istituto Nazionale Tumori di Milano, Via Venezian, 1-20133 Milan, Italy; 4 Unità di Medicina Nucleare, Istituto Nazionale Tumori di Milano, Via Venezian, 1-20133 Milan, Italy; 5 Servizio di Anatomia Patologica e Istologia, Istituto Nazionale Tumori di Milano, Via Venezian, 1-20133 Milan, Italy; 6 Servizio di Radiodiagnostica, Istituto Nazionale Tumori di Milano, Via Venezian, 1-20133 Milan, Italy; 7 Servizio Immunotrasfusionale. Istituto Nazionale Tumori di Milano, Via Venezian, 1-20133 Milan, Italy

**Keywords:** neuroblastoma, sequential hemi-body irradiation, autologous stem cell transplantation

## Abstract

The aim of the present study was to evaluate the effectiveness of two consecutive nonrandomised treatment programs applied between 1989 and 1999 at the Istituto Nazionale Tumori of Milan in an unselected cohort of 59 children over the age of one with stage 4 neuroblastoma. Both treatment programs consisted of two phases, the induction of the remission phase and the consolidation phase. The induction of the remission phase consisted of intensive chemotherapy, and remained the same throughout the study period. The consolidation phase consisted of sequential hemi-body irradiation (HBI) (10 Gy per session, 6 weeks apart) in the first period (1988–June 1994) and sequential high-dose cyclophosphamide, etoposide, mitoxantrone+L-PAM and autologous haemopoietic stem cell transplantation in the second (July 1994–1999). Intention-to-treat analysis revealed a significantly better outcome for patients treated with the second program, the 5-year event-free survival probability being 0.12 for program 1 and 0.31 for program 2 (*P*=0.03). This finding led us to conclude that sequential HBI is useless as consolidation treatment. The high-dose chemotherapy adopted in the second program enabled a proportion of patients to obtain long-term survival but, since the clinical results remain unsatisfactory, new treatment strategies are warranted.

In the past two decades, the prognosis for patients with stage 4 neuroblastoma over the age of 1 year has progressively changed, with a weak but significant improvement in clinical results (Goldsby and Matthay, 2004). This slight improvement can be related to the greater and greater aggressiveness of the treatment: intensified chemotherapy increases the percentage of clinical remissions, the introduction of megatherapy helps to eradicate resistant clones, and the addition of ‘maintenance’ therapy is expected to control any minimal residual disease (Cheung *et al*, 1998; Pession *et al*, 1998; Garaventa *et al*, 1999; Matthay *et al*, 1999; Pinkerton *et al*, 2000). The use of aggressive surgery and radiotherapy to the primary tumour site may also contribute to reducing the risk of local relapse (Kushner *et al*, 2001; Haas-Kogan *et al*, 2003; La Quaglia *et al*, 2004). Nowadays, the majority of clinical trials on advanced neuroblastoma are organised according to the sequence: ‘induction’ of remission with chemotherapy and local treatment at the primary tumour site, ‘consolidation’ with megatherapy, and ‘maintenance’ therapy (Matthay *et al*, 1999; Berthold and Hero, 2000; De Bernardi *et al*, 2003). The number of studies focusing on the impact of each treatment modality within this arrangement is still limited, however, and the studies on this issue with a randomised design are rarer still (Ladenstein *et al*, 1994; Pearson *et al*, 1994; Matthay *et al*, 1999; Berthold and Hero, 2000; Pritchard *et al*, 2005).

In the present study, we describe a mono-institutional experience of two consecutive nonrandomised treatment programs comprising an identical induction phase, followed by two different strategies for the consolidation phase, that is, sequential fractionated hemi-body irradiation (HBI) in the first case and sequential high-dose chemotherapy plus autologous haemopoietic stem cell transplantation in the second. Our aim was to explore the impact of these two strategies on outcome.

## PATIENTS AND METHODS

All children over the age of 1 year with previously untreated stage 4 neuroblastoma diagnosed from 1989 to 1999 at the Istituto Nazionale Tumori of Milan were prospectively enrolled in the institutional treatment programs.

Diagnosis, staging and response to therapy were evaluated according to the International Neuroblastoma Staging System and the International Neuroblastoma Response Criteria (Brodeur *et al*, 1993). The diagnosis was based on a histological examination or, in some cases, on the documentation of an unequivocal bone marrow infiltration. The initial evaluation included CT or MRI of the primary tumour, 131-I-mIBG scan, TC-99-MDP scan, bilateral bone marrow biopsy and aspirate, serum levels of LDH, neuron-specific enolase and ferritin, and urinary concentrations of vanylmandelic and homovanillic acids. Response to treatment was assessed after each of the two steps in the treatment strategy (induction and consolidation).

During the study period (1989–1999), two different, non-randomised treatment programs were adopted (Table 1), both approved by the local Ethical and Scientific Board. The intention was to treat every new child over the age of one admitted to our institution with previously untreated stage 4 neuroblastoma. A stopping rule for toxic deaths was set for both treatment programs. The first program (program-1), applied between January 1989 and June 1994, consisted of an ‘induction of remission’ phase with intensive chemotherapy and a ‘consolidation of remission’ phase with sequential fractionated HBI. Briefly, the induction phase consisted of eight cycles of vincristine+cisplatinum+etoposide+epi-adriamycin alternated at 4-week intervals with vincristine+cisplatinum+etoposide+ifosfamide. In each patient, the doses of all drugs except vincristine had to be scaled up by 20% at each subsequent cycle if the nadir after the previous cycle coincided with WBC ⩾1000 cumm^−1^ and/or platelets ⩾70 000 cumm^−1^. The doses had to be scaled down by 20% if the nadir coincided with WBC <1000 and/or platelets <70 000 cumm^−1^; the minimal doses given were 100%. At 3 weeks after ending the induction, patients with an objective response (complete, very good, or partial remission) began the consolidation phase. In program-1, this consisted of two consecutive sessions of HBI. The upper and lower body halves were irradiated, with a 6-week interval between the two sessions, using a fractionated technique described elsewhere (Lombardi *et al*, 1989): 10 Gy, as midplane dose, were delivered in five daily fractions, 2 Gy day^−1^, through two lateral opposite portals at a dosage of 0.2 Gy min^−1^ by a 15 MeV linear accelerator. Program-1 had two aims, that is, to reach an objective response rate higher than the 60% achieved by the previous treatment program (Lombardi *et al*, 1989), and to evaluate the impact of HBI on clinical outcome.

The second program (program-2) was adopted between July 1994 and December 1999. This program only differed from the previous one as regards the consolidation phase, which consisted of a sequence at 3-week intervals of cyclophosphamide 7 g sqm^−1^, etoposide 2 g sqm^−1^, mitoxantrone 60 mg sqm^−1^+melphalan 210 mg sqm^−1^, followed by autologous peripheral blood stem cell transplantation (Table 1). Stem cells were collected from peripheral blood using G-CSF 10 *μ*g kg^−1^ day^−1^ as of day 4 after administering cyclophosphamide up until the day of leukapheresis. If this target was not reached after cyclophosphamide (at least 3 × 10e6 CD34+cells kg^−1^), an additional leukapheresis was performed after etoposide, using G-CSF at the same dose as after cyclophosphamide. The aim of program-2 was to evaluate the impact of this consolidation phase on clinical outcome.

In both programs 1 and 2, surgical resection of the primary tumour was on an individual basis and considered as part of the induction of remission phase. In addition, ‘local’ treatment with radiotherapy was not specified by the treatment plan, and no further treatment was planned after the end of the consolidation phase. Supportive care policies did not change substantially during the study period, with the exception of the use of G-CSF, from 1996 onwards, in cases of febrile neutropenia and documented infection.

In the present study, we describe our mono-institutional experience with the two programs described above. The primary goal of the present study was to assess event-free survival (EFS) and survival (S) probabilities for the two groups of patients enrolled on programs 1 and 2 using an intention-to-treat analysis. Event-free survival was calculated from the first day of treatment up until progression or relapse, or death due to toxicity. Survival was calculated from the first day of treatment until death. EFS and S distributions were estimated using the Kaplan–Meier method, and compared with the log-rank test. To evaluate the homogeneity of the characteristics of the patients enrolled in programs 1 and 2, the frequency distribution of different variables in the two groups was compared using the *χ*^2^ test for: sex, age, site of primary tumour, LDH level, serum ferritin, serum NSE, AVM/HVA dosage, cumulative % of drug dosages delivered during induction (⩾120 *vs* <120% of the initial dose), surgery on the primary tumour site.

## RESULTS

All 59 consecutive children over the age of 1 year with stage 4 neuroblastoma at onset observed at the Istituto Nazionale Tumori of Milan during the study period were enrolled. In total, 25 joined program-1 and 34 joined program-2. In all cases, the diagnosis, treatment and follow-up were completed at the Istituto Nazionale Tumori.

The patients' demographics and clinical characteristics are shown in Table 2. The M/F ratio was 1.3, and the median age at diagnosis was 3 years (range 1–18). The site of primary tumour was the retroperitoneum/adrenal gland in the majority of patients (85%). Skeleton and bone marrow were the most frequent sites of metastases (85 and 72%, respectively), followed by distant lymph nodes (37%), liver (14%), orbitae (14%), central nervous system (8%) and lungs (3%). The distribution of clinical variables at onset, the drug doses given, the number of patients who underwent surgery on the primary tumour site (Table 2), and the response to induction treatment (Table 3) were similar in the two groups.

All patients received at least two cycles of induction therapy and were evaluable for response. The maximal response during induction was as follows: complete remission, 10; very good partial remission, 10; partial remission, 33; no response (stable disease or progression of disease) 6. In all, the percentage of responders was 89%. Six of the patients with partial remission experienced progression of disease during the induction treatment, however, so 47 patients concluded the induction phase and entered the consolidation phase. Two additional patients – one after the first HBI session in program-1, and one after high-dose VP16 in program-2 – had progression of disease during the consolidation phase. Thus, 45 patients completed the entire treatment plan, 18 out of 25 (72%) in program-1 and 27 out of 34 (79%) in program-2. Consolidation treatment determined a further response in two out of 19 (11%) after HBI in program-1 and in 16 out of 27 (59%) after high-dose therapy in program-2 (Table 3). As for the timing of the treatments, 62% of cases concluded the treatment program as scheduled, or with a delay <15 days, while the remaining 38% of cases had an overall delay ⩾15 days (range 15–45). The percentage of cases with a delay of the timing of the treatment in program-1 and program-2 were superimposable (data not shown). For both programs, acute toxicity was mainly haematological. There were no toxic deaths or treatment interruptions due to severe adverse events.

**Figure 1:**
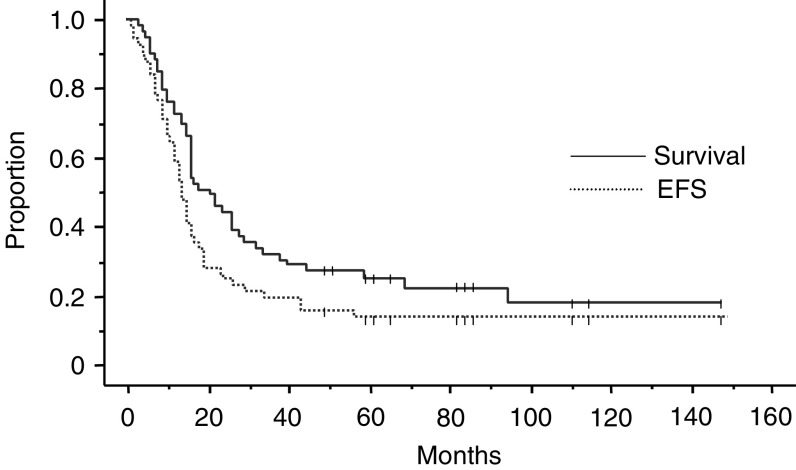
Event-free survival (EFS) and survival of the entire series.

**Figure 2:**
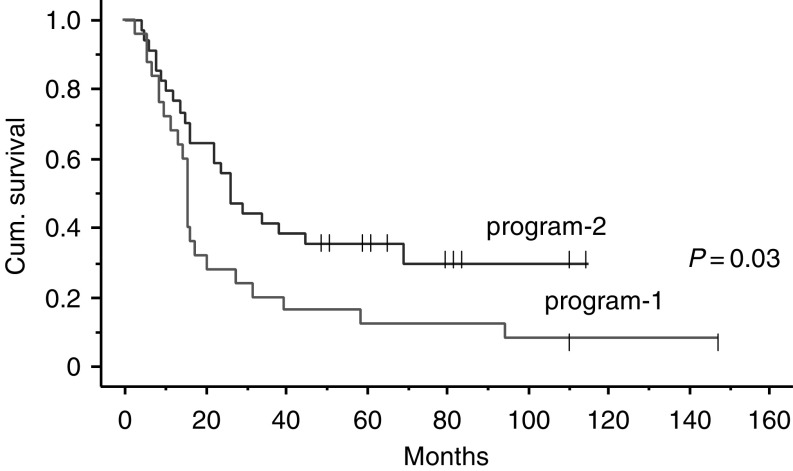
Survival by treatment program.

Among the patients who concluded the treatment program, 31 relapsed a median 16 months after diagnosis (range 9–66). The relapse pattern was: metastatic spread, 21 (68%); metastatic spread plus local relapse, eight (26%); isolated local relapse, two (6%). None of the patients with nonresponse or progression of disease survived after second-line therapy. In all, 46 out of 59 patients died (22 in program-1 and 24 in program-2). The median follow-up for the entire series at the time of the current analysis (as at June 2004) was 62 months (range 51–164). The 5-year EFS and 5-year S probability for the entire series was 0.18 and 0.25, respectively (Figure 1). Analysing the outcome according to the treatment program adopted revealed a significant difference between the two: the 5-year EFS probability was 0.12 for program-1 and 0.31 for program-2 (*P*=0.03); the 5-year S probability was 0.12 for program-1 and 0.35 for program-2 (*P*=0.03) (Figure 2).

## DISCUSSION

Total-body irradiation (TBI) has been widely used in the treatment of advanced NB, mainly as a consolidation strategy, followed by haemopoietic stem cell transplantation (Ladenstein *et al*, 1994; Matthay *et al*, 1999). Alternative methods of TBI had also been applied in NB, consisting of cyclic low-dose TBI (D'Angio and Evans, 1983) or sequential HBI in combination with chemotherapy (Helson *et al*, 1981). The results of treatment with cyclic irradiation or HBI and concomitant chemotherapy were similar to those obtainable by chemotherapy alone, and were complicated by a more severe toxicity, so these modalities were abandoned. Sequential HBI in advanced NB was also used at the Istituto Nazionale Tumori of Milan, but in a different way, that is, HBI was given as a consolidation strategy after the remission phase, and the results of this experience suggested that fractionated HBI for consolidation after chemotherapy might have a favourable effect on outcome (Lombardi *et al*, 1989). The promising results of this experience led us to design the program-1 described in the present study in 1989, in which the induction phase with a combination of five drugs in rising doses led to a response rate of 89%, a result comparable with those obtained in other trials (Berthold and Hero, 2000; Pinkerton *et al*, 2000; Goldsby and Matthay, 2004). Despite the use of intensive chemotherapy, no toxic deaths were encountered and none of the patients had to interrupt the treatment due to toxicity. The clinical outcome of patients treated with program-1 remained poor, however, since the majority had recurrent disease within 24 months of diagnosis despite the high percentage of responders in the induction phase. This led us to conclude that sequential HBI was useless as a consolidation treatment in advanced NB.

Program-2 was designed to try to improve on the results obtained by program-1 by modifying the consolidation phase. Our aim was to explore the role of high-dose sequential cyclophosphamide, etoposide and mitoxantrone+melphalan in erasing residual neoplastic clones proving resistant to the induction phase. Using this schedule, different drugs known to be active in NB were used, with the exception of mitoxantrone, whose activity in NB had been demonstrated *in vitro* (Fulda *et al*, 1995) but had never been explored *in vivo* (Kushner *et al*, 1990; Pinkerton, 1991; Berthold and Hero, 2000). The consolidation treatment adopted in program-2 led us to support the activity of megatherapy in advanced NB, on the grounds of two arguments. The first is the further response obtained with high-dose therapy after the induction therapy in a consistent number of cases – notably higher than the response obtained with HBI. The second is the 5-year survival probability of patients treated with program-2, which was 0.35 – significantly better than for patients treated with program-1.

In the present study, only a small number of patients had surgery on the primary tumour site so we cannot draw any conclusions concerning the impact of a ‘local’ treatment on the survival probability in this series. Since surgery and radiotherapy on the primary tumour site have been shown to contribute to controlling local relapses in stage 4 NB (Kushner *et al*, 2001; Haas-Kogan *et al*, 2003; La Quaglia *et al*, 2004), it may be that adding a ‘local’ treatment with surgery plus radiotherapy in an extended way to program-2 might further improve the results we obtained.

We are naturally aware that the limited number of patients enrolled over a lengthy study period and the mono-institutional setting could constitute a bias of our study, and that the statistical results should be interpreted with caution as there might be additional unforseen bias due to the nonrandom design of the treatment programs applied. Furthermore, the possibility that the use of G-CSF could have concurred in the better outcome of program-2 cannot be excluded for certainty. In fact, in the two programs no differences were recorded as to the timing of drug administration schedule and no treatment discontinuation due to acute severe toxicity were recorded in either program. This study included an unselected cohort of patients, the results are described as intention-to-treat, and the survival probability is calculated on the strength of a long follow-up. The use of the same induction treatment throughout the study and the comparable clinical characteristics of the two groups of patients strongly suggest the superiority of the therapeutic results of consolidation treatment with sequential high-dose chemotherapy and autologous stem cell transplantation. Our experience can be added to the limited number of other papers comparing different consolidation strategies after a common induction phase, thus making the results of these strategies fully comparable. These studies evaluated megatherapy *vs* continuing intensive chemotherapy (Matthay *et al*, 1999), or maintenance chemotherapy (Berthold *et al*, 1990; Castel *et al*, 2001), or no treatment (Pritchard *et al*, 2005). Combined with the other experiences including megatherapy, they reliably support the conviction that this consolidation treatment modality offers an advantage in terms of survival probability (McCowage *et al*, 1995; Kushner *et al*, 2001; Frappaz *et al*, 2002; Kaneko *et al*, 2002; Kletzel *et al*, 2002; De Bernardi *et al*, 2003).

In spite of all the possible bias, the present study supports the claim that patients with stage 4 NB over the age of 1 year with a tumour responding to initial intensive chemotherapy can benefit from high-dose chemotherapy and haemopoietic stem cell rescue. We are aware that this type of study is not the best way forward and that important clinical questions should nowaday be answered by randomised trials conducted on a multicentre basis with international cooperation. Any chances of improving on the clinical results will obviously come also from new insights on the biology of neuroblastoma and the availability of new active molecules.

## Figures and Tables

**Table 1:** Treatment programs for stage 4 neuroblastoma over the age of 1 year, 1989–1999

											**Consolidation phase**		
**Induction phase**											**Program-1 (January 1989–June 1994)**		
Course	A	B		A	B	A	B	A	B	S	1st HBI	2nd HBI	
	↓	↓		↓	↓	↓	↓	↓	↓	↓	↓	↓	
Week	**0**	**4**		**7**	**10**	**13**	**16**	**19**	**22**		**26**	**32**	
													
											**Program-2 (July 1994–December 1999)**		
											HDCTX	HDVP16	MGT+autograft
											↓	↓	↓
											**26**	**29**	**32**

Course A=vincristine day 1; etoposide+cisplatinum+epirubicin days 1–4.

Course B=vincristine day 1; etoposide+cisplatinum+ifosfamide days 1–4.

S=surgery on the primary tumour site, on individual basis.

HBI=hemi-body irradiation.

HDCTX=high-dose cyclophosphamide; HDVP16=high-dose etoposide; MGT+autograft=high-dose mitoxantrone (day −5)+melphalan (day −2)+autologous stem cell transplantation (day 0).

**Table 2:** Clinical characteristics at onset and treatment given before consolidation treatment

	**Program-1**		**Program-2**	
	**Total=25**	**%**	**Total=34**	**%**
*Sex*				
Male	14	56	19	56
Female	11	44	15	44
				
*Age ranges (months)*				
12–24	9	36	11	32
25–216	16	64	23	68
				
*Primary tumour site*				
Retroperitoneum/adrenal gland	22	88	27	79
Other sites	3	12	7	21
				
*Bone marrow infiltration*				
Absent	6	24	8	23
Present	19	76	23	77
				
*Bone metastases*				
Absent	5	20	4	12
Present	20	80	30	88
				
*LDH (56 evaluated)*				
<2*n*	9	37	12	37
⩾2*n*	15	63	20	63
				
*NSE (54 evaluated)*				
<100 ng ml^−1^	10	43	12	39
⩾100 ng ml^−1^	13	57	19	61
				
*Ferritin (47 evaluated)*				
<143 ng ml^−1^	5	25	9	33
⩾143 ng ml^−1^	15	75	18	67
				
*VMA/HVA ratio (44 evaluated)*				
<1	10	62	18	64
⩾1	6	38	10	36
				
*Mean dose of chemotherapy during induction phase*				
<120%	11	58	19	70
⩾120%	8	42	9	30
				
*Surgery on primary tumour site*				
No	17	84	19	71
Yes	2	16	9	29

Abbreviations: LDH=lactate dehydrogenase; NSE=neuron-specific enolase; VMA=vanylmandelic acid; HVA=homovanillic acid.

47 patients who concluded the induction phase.

The distribution of clinical variables at onset, the drug doses given, the number of patients who underwent surgery on the primary tumour site were similar in the two groups (*P*: NS for all variables).

**Table 3:** Response to therapy by treatment program

	**Program-1**		**Program-2**	
	**No. of patients**	**%**	**No. of patients**	**%**
*After induction phase (59 patients)*				
Complete remission	4	16	6	18
Very good partial remission	5	20	5	15
Partial remission	11	44	16	47
No response/progression	5	20	7	20
				
*After consolidation phase (47 patients)*				
Complete remission	4	20	16	59
Very good partial remission	7	35	8	30
Partial remission	8	40	2	7
No response/progression	1	5	1	4
